# Exploratory assessment of right ventricular structure and function during prolonged endurance cycling exercise

**DOI:** 10.1186/s44156-023-00035-8

**Published:** 2023-12-20

**Authors:** Rachel N. Lord, Zoe H. Adams, Keith George, John Somauroo, Helen Jones, David Oxborough

**Affiliations:** 1 Centre for Health Activity and Wellbeing Research, Cardiff Metropolitan University, Cyncoed Campus, Cyncoed Road, Cardiff, CF23 6XD UK; 2 Research Institute for Sport and Exercise Sciences, Liverpool, UK; 3 Countess of Chester Hospital, NHS Trust, Chester, UK

**Keywords:** Endurance, Exercise, Ultrasound, Right ventricular function

## Abstract

**Background:**

A reduction in right ventricular (RV) function during recovery from prolonged endurance exercise has been documented alongside RV dilatation. A relative elevation in pulmonary artery pressure and therefore RV afterload during exercise has been implicated in this post-exercise dysfunction but has not yet been demonstrated. The current study aimed to assess RV structure and function and pulmonary artery pressure before, during and after a 6-h cycling exercise bout.

**Methods:**

Eight ultra-endurance athletes were recruited for this study. Participants were assessed prior to exercise supine and seated, during exercise at 2, 4 and 6 h whilst cycling seated at 75% maximum heart rate, and post-exercise in the supine position. Standard 2D, Doppler and speckle tracking echocardiography were used to determine indices of RV size, systolic and diastolic function.

**Results:**

Heart rate and RV functional parameters increased from baseline during exercise, however RV structural parameters and indices of RV systolic and diastolic function were unchanged between in-exercise assessment points. Neither pulmonary artery pressures (26 ± 9 mmHg vs 17 ± 10 mmHg,* P* > 0.05) nor RV wall stress (7.1 ± 3.0 vs 6.2 ± 2.4, *P* > 0.05) were significantly elevated during exercise. Despite this, post-exercise measurements revealed RV dilation (increased RVD1 and 3), and reduced RV global strain (− 21.2 ± 3.5 vs − 23.8 ± 2.3, *P* = 0.0168) and diastolic tissue velocity (13.8 ± 2.5 vs 17.1 ± 3.4, *P* = 0.019) vs pre-exercise values.

**Conclusion:**

A 6 h cycling exercise bout at 75% maximum heart rate did not alter RV structure, systolic or diastolic function assessments during exercise. Pulmonary artery pressures are not elevated beyond normal limits and therefore RV afterload is unchanged throughout exercise. Despite this, there is some evidence of RV dilation and altered function in post-exercise measurements.

## Background

Exercise induced changes in cardiac structure and function during recovery from prolonged endurance exercise have been well documented and are commonly referred to as exercise induced cardiac fatigue. Recent research has focused on the right ventricle (RV) and a reduction in both systolic and diastolic RV function has been reported from prolonged endurance exercise of several modalities [5, 21, 28, 31]. Dilatation of the RV is also evident following greater duration endurance exercise suggestive of a possible overload to the right heart [15, 25, 30]. Endurance cycling, the modality used in the current study, is among those associated with reduced RV function, as assessed by echocardiographic [32, 33] and cardiac MRI techniques [5].

The majority of studies investigating cardiac fatigue have focused on comparisons between pre-race and recovery measures, with a void in the literature focusing on the in-exercise response of the RV. There are a limited number of studies assessing cardiac function during exercise. An increase in RV systolic tissue velocity has been demonstrated during submaximal cycling exercise [11, 35] with results indicating a physiological linear response between increasing exercise intensity and increasing contractility. However, there are no data available to characterise the diastolic functional response of the RV during exercise. Using more novel techniques, Goebel et al. [11], Tan et al. [35] and La Gerche et al. [14] applied strain imaging to the RV during submaximal cycling, stress echo and progressive maximal cycling respectively and reported an increase in RV strain. Banks et al. [2] report a reduction in RV strain and strain rates at 150 min of running. However, participants in this study were transferred from the treadmill to echo bed and stopped exercising for the duration of the assessment. Heart rate drops significantly on exercise cessation and the echocardiograms were obtained with subjects in a supine as opposed to upright position therefore impacting on load and not giving a true reflection of in-exercise RV function. The equivocal results and short duration exercise stimuli employed make firm conclusions about the in-exercise RV response challenging.

Several possible mechanisms have been suggested to explain cardiac fatigue in the RV. These include beta-adrenergic receptor down-regulation [2] and subsequent reduction in contractility; inflammation or biomarker release [18]; or an elevated RV afterload secondary to a disproportionally elevated pulmonary compared to systemic pressure [17]. The pulmonary circulation has a limited capacity for vasodilation and therefore pulmonary artery pressures (PAP) during exercise are relatively higher when compared to the systemic circulation and can reach values indicative of pulmonary hypertension during prolonged endurance exercise [13]. La Gerche et al. have quantified this disproportionate RV exercise stroke work in a semi-supine progressive cycling exercise fitness test and reported significantly elevated PAP up to 61 mmHg during exercise. The use of cardiac MRI to derive RV end systolic volume and echocardiography to estimate PAP does not afford simultaneous assessment of the components that RV wall stress is calculated from and although the authors attempt to correct for the two techniques involved in the measurement, there is a large potential for error. The concurrent assessment of RV structure and function alongside pulmonary artery pressures using echocardiography may aid the understanding of temporal exercise induced changes in a prolonged upright exercise bout. The aim of this study is therefore to build on previous research and assess RV structure and function and pulmonary artery pressures before, at 2-h intervals during, and following a 6-h cycling exercise bout. We hypothesised that PAP would be elevated during prolonged strenuous cycling exercise and that RV dysfunction and structural enlargement would be present post-exercise.

## Methods

### Sample population

Eight well trained male ultra-endurance athletes (Body mass 77.8 ± 11 kg, height 179 ± 6 cm, BP 136/88 mmHg, age 40 ± 7 years, VO_2_ max 51.9 ± 10 ml kg min^−1^) gave written informed consent to participate in this study. Participants self-reported: no known cardiovascular disease, no prescribed medications and no comorbidities or family history of cardiovascular disease. Ethical approval was granted by the University Ethics Committee.

### Protocols

Participants underwent a maximal oxygen uptake test (Oxycon, Care Fusion, Hoechberg, Germany and SRM bike, Jülich, Germany) at 30 W increments every 3 min to determine their maximum heart rate on a separate day to the 6-h cycling session. Participants were requested to avoid vigorous training, alcohol, and caffeine for a minimum of 24 h prior to the assessment. On the day of the cycling session, systolic and diastolic blood pressure was assessed prior to and immediately after exercise using standard auscultation (Dinamap pro, GE Healthcare, Horten, Norway). Echocardiography assessments were done prior to the exercise session in both supine and seated positions; at 2-, 4- and 6-h intervals during the exercise bout (seated cycling); and in the supine position immediately post exercise. All images were acquired using a commercially available ultrasound system (Vivid Q, GE Medical, Horten, Norway) with a 1.5–4 MHz phased array transducer. In-exercise images were obtained by a single experienced sonographer (DLO) with the participant on their own road cycling bike fixed to a turbo trainer device cycling at 75% maximum heart rate. Images were recorded to DVD in raw DICOM format and data were analysed offline by a single experience sonographer (RNL) using commercially available software (EchoPac version 7, GE Medical, Horten, Norway). A minimum of three cardiac cycles were averaged for all peak indices.

### Conventional 2D, Doppler and tissue Doppler echocardiography

The RV was assessed in accordance with British Society of Echocardiography guidelines [38] providing structural indices at the outflow tract (RVOT_plax_, RVOT_1_, and RVOT_2_) and at the inflow (RVD_1_, RVD_2_, RVD_3_). RV diastolic area (RVAd) and systolic area (RVAs) were measured, and the fractional area change calculated (RVFAC). A pulsed wave tissue doppler imaging (TDI) sample positioned at the tricuspid annulus allowed the assessment of RV S’, E’ and A’ myocardial velocities. Right ventricular systolic pressure (RVSp) was derived from the tricuspid regurgitant jet (TR velocity) using continuous wave Doppler. The regurgitant signal was improved for resting and exercise measurements using agitated saline administered via a three way stop cock cannula inserted into the antecubital vein as previously described [1]. This technique has been shown to improve the accuracy of both resting and exercising assessments of pulmonary artery pressures [20] and agitated saline was therefore administered to our participants immediately prior to echocardiographic assessment at rest, at 2, 4 and 6 h intervals into the cycling session and during post-exercise assessment. Pulmonary artery systolic pressure (PASP) was calculated as (PASP (mmHg) = RVS_p_ + 5 mmHg)_._ For RV end-systolic wall stress, Laplace’s law was used to calculate according to the formula Pr/2 h where P (pressure) was quantified as PASP, r (radius) was calculated using the formula r = ${0.620(\mathrm{RVSa})}^\frac{1}{3}$, assuming spherical geometry as previously described [24] and h was quantified as RV wall thickness.

### 2D myocardial speckle tracking

Based on a previous study by our group suggesting limited feasibility, comparability and reliability of myocardial speckle tracking to derive RV longitudinal strain above 50% maximum heart rate, we have only assessed RV strain at rest pre and post-exercise [23]. A modified apical 4 chamber image with lateral transducer movement was acquired for assessment of the RV strain. For all images the system was optimised as previously described [30]. Offline analysis allowed the assessment of peak global longitudinal RV strain (calculated as an average of 6 myocardial segments from base to apex of the RV free wall and septum) and RV free wall strain calculated as an average of the 3 myocardial basal, mid and apical segments of the RV free wall.

### Statistics

Echocardiographic data were analysed for normality of distribution using a Shapiro–Wilk test. Seated baseline and peak data at 2, 4 and 6 h of exercise were compared using a one-way repeated measures ANOVA. Pre- to post-exercise supine data were compared using a paired samples T-test. All statistical tests were performed using commercially available software (IBM SPSS version 22) and statistical significance was set as *P* < 0.05.

## Results

### Exercise responses

Heart rate was significantly higher during exercise at 2, 4 and 6 h compared to baseline (*P* = 0.04, 0.04 and 0.03 respectively, Table 1). There was a significant increase (*P* = 0.003) in TAPSE from baseline to 4 h and RV S’ and RV A’ were also significantly elevated (*P* = 0.001, 0.015 and 0.006 and > 0.001 respectively, Table 1) from baseline at 2, 4 and 6 h into exercise. There were no significant differences in RVFAC, TR velocity, PASP or RV wall stress from baseline to in-exercise measures (*P* > 0.05, Table 1). There were also no significant differences in RV structural parameters RVOTplax, RVOT1, RVOT2, RVD1, RVD2, RVD3, RVAd and RVAs from baseline to in-exercise assessment points (*P* > 0.05, Table 1).

**Table 1:** Right ventricular structural and functional indices during exercise

Variable	Baseline	2-h	4-h	6-h
Heart rate (beats.min^−1)^	60 ± 10	131 ± 12^ɣ^	134 ± 16*	146 ± 27^ʘ^
RVOT_plax_ (mm)	30 ± 3	31 ± 2	32 ± 3	33 ± 3
RVOT1 (mm)	32 ± 4	33 ± 6	34 ± 4	33 ± 5
RVOT2 (mm)	22 ± 3	22 ± 3	24 ± 2	23 ± 2
RVD1 (mm)	46 ± 5	47 ± 3	49 ± 5	47 ± 5
RVD2 (mm)	28 ± 5	28 ± 4	29 ± 3	30 ± 4
RVD3 (mm)	85 ± 10	89 ± 6	90 ± 4	89 ± 4
RVAd (cm^2^)	26.4 ± 4.7	27.0 ± 2.7	28.5 ± 3.8	27.9 ± 3.1
RVAs (cm^2^)	12.9 ± 1.7	13.3 ± 2.5	13.2 ± 1.6	13.8 ± 1.8
RVFAC (%)	50.6 ± 6.0	50.9 ± 5.4	53.5 ± 6.2	50.5 ± 6.2
TAPSE (mm)	28 ± 3	32 ± 2	35 ± 4*	32 ± 3
RV S’ (cm/s)	17 ± 3	34 ± 6^ɣ^	31 ± 7*	32 ± 5^ʘ^
RV E’ (cm/s)	17 ± 41	20 ± 4	20 ± 6	20 ± 9
RV A’ (cm/s)	13 ± 3	29 ± 4^ɣ^	34 ± 3*	34 ± 2^ʘ^
PASP (mmHg)	17 ± 10	20 ± 9	21 ± 9	26 ± 9
TR velocity (m/s)	1.9 ± 0.5	2.1 ± 0.2	2.1 ± 0.6	2.4 ± 0.2
RV wall stress (kdynes/cm^2^)	6.2 ± 2.4	5.8 ± 3.4	5.5 ± 2.2	7.1 ± 3.0

One-way repeated measures ANOVA, statistical significance indicated as ɣ = baseline to 2 h, ^*^ = baseline to 4 h, ʘ = baseline to 6 h. Data are mean ± SD, N = 8, except for RVOT_plax_ where n = 7. Baseline data were obtained with participants sat upright on the bike

### Pre- and post-exercise comparison

**Fig. 1:**
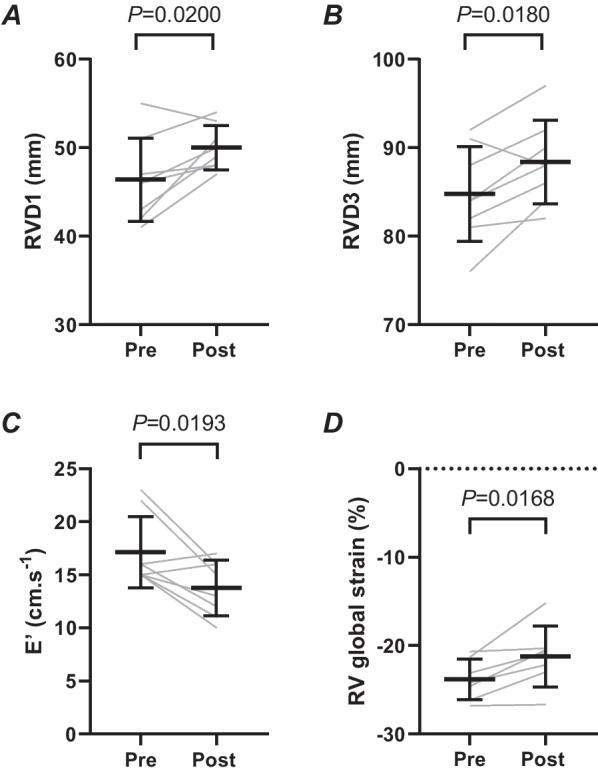
Right ventricular dimensions (**A**, **B**), early diastolic tissue velocity (**C**), and global strain (**D**) pre- and post-exercise. Data are mean ± SD, paired-T test. N = 8 for A–C and N = 7 for D

Body mass (77.8 ± 11 and 78 ± 11.3 kg) and diastolic blood pressure (78 ± 8 and 70 ± 5 mmHg) were unchanged pre- to post-exercise (*P* > 0.05). Heart rate (HR) was significantly increased post-exercise (75 ± 11 beats min^−1^) compared to pre-exercise (55 ± 10 beats min^−1^, *P* = 0.001) and systolic blood pressure was significantly reduced (*P* = 0.04) pre- to post-exercise (136 ± 13 to 123 ± 10 mmHg). Structural parameters RVOT_plax_, RVOT 1 and 2, RVD2, RVAd, and RVAs were not significantly different pre- to post-exercise (*P* > 0.05, Table 2). In contrast, RVD1 and RVD3 were significantly larger post-exercise versus baseline (RVD1 50.0 ± 2.5 mm vs 46.4 ± 4.7, *P* = 0.02; RVD3 88.4 ± 4.7 vs 84.8 ± 5.4 mm, *P* = 0.02; Fig. 1A, B). With respect to functional parameters, TAPSE, S’, A’, TR velocity, PASP, RV wall stress and RV free wall strain were not significantly different pre- to post-exercise (*P* > 0.05, Table 2). However, RV global strain (− 21.2 ± 3.5 vs − 23.8 ± 2.3, *P* = 0.02) and E’ (13.8 ± 2.5 vs 17.1 ± 3.4, *P* = 0.02) were significantly reduced post-exercise versus baseline (Fig. 1C, D).

**Table 2:** Right Ventricular Structural and Functional Indices pre- and post-exercise

Variable	Pre-exercise	Post-exercise	*P* value
RVOT_plax_ (mm)	35 ± 3	35 ± 5	0.57
RVOT1 (mm)	36 ± 6	36 ± 6	0.44
RVOT2 (mm)	26 ± 2	27 ± 2	0.48
RVD2 (mm)	33 ± 3	33 ± 3	0.64
RVDa (cm^2^)	28.0 ± 3.6	31.4 ± 4.5	0.11
RVSa (cm^2^)	14.6 ± 2.6	15.4 ± 2.1	0.23
RVFAC (%)	48 ± 5	51 ± 5	0.26
TAPSE (mm)	27 ± 5	28 ± 5	0.57
RV S’ (cm/s)	16 ± 2	17 ± 4	0.81
RV A’ (cm/s)	16 ± 3	16 ± 3	0.94
PASP (mmHg)	22 ± 7	20 ± 3	0.26
TR velocity (m/s)	2.2 ± 0.8	2.1 ± 0.3	0.45
RV Wall Stress (kdynes/cm^2^)	6.4 ± 2.7	5.8 ± 1.8	0.33
RV free wall strain (%)	− 28.9 ± 3.2	− 28.2 ± 3.9	0.60

Paired T-test, mean ± SD. N = 8, except for RVOT_plax_ and RV free wall strain where n = 7. Pre-exercise data were obtained in the supine position

## Discussion

This study investigated the factors contributing to reduced RV function after endurance exercise, using echocardiographic assessment of RV function in athletes before, during, and after a 6-h cycling bout. Contrary to our hypothesis that increased PAP and RV afterload would occur during exercise and precede a reduction in RV function, we measured no PAP or afterload changes during exercise, despite evidence of RV dilation and reduced systolic and diastolic function post-exercise.

### RV dilation and dysfunction post-exercise

A dilation of the RV and reduction in systolic and diastolic function after ultra-endurance exercise has been reported by several groups using a range of echocardiographic techniques and indices [8, 15, 16, 26, 30]. Increased RV size, reduced RV lateral wall myocardial annular velocities, and lower RV strain suggest reduction in myocardial relaxation and contraction post-exercise. These changes are generally transient, with most parameters returning to baseline levels within days [15, 16]. There is some evidence that RV strain rate remains reduced 6–11 days after the end of the exercise stimulus [15], although the time to recovery remains unclear.

The commonly accepted potential mechanism responsible for these RV changes centers on an elevated PASP during exercise resulting in a disproportionate afterload for the RV during exercise [17, 30]. Increased PAP in endurance exercise has been demonstrated by both echocardiographic [28] and direct right heart catheterisation [4] methods. Indeed, two meta-analyses have supported the notion that PAP often exceeds 30 mmHg during exercise and is dependent, in part, on age and exercise intensity [9, 13]. Moreover, trained athletes have a greater PAP both at rest [7] and during exercise compared to untrained individuals [6, 9, 19, 36], likely secondary to an increased RV stroke volume and inability of the pulmonary vasculature to vasodilate to the same degree as the systemic vasculature. Reduced RV function with a concurrent increase in PASP was reported by Neilan et al. but not by Buchan et al., although this discrepancy could be attributed to the much shorter exercise stimulus duration used in the latter study. Others have previously shown a decline in RV function with a reduction in PASP [15], whilst we previously found no change in PASP [22]. This variability could result from the measurements being taken after the exercise stimulus had finished, with the participant supine, and from the variable relative exercise intensity that participants exercised at. Thus, we expected that the current data would demonstrate detectable increases in PASP and RV afterload *during* exercise. Interestingly, although a non-significant 50% increase in PASP was evident 6-h into exercise, RV wall stress (as a surrogate of afterload) was not elevated at 2-, 4- or 6-h into the 6-h exercise bout. Despite this, a post-exercise dilation of the RV alongside a reduction in both systolic and diastolic function was evident. Thus, post-exercise RV dysfunction occurred in the setting of detectable increases in PASP but not RV afterload during exercise, raising the possibility that elevated PAP may partially explain RV dysfunction after 6-h of prolonged cycling. Alternative mechanisms may therefore contribute to some degree of RV dysfunction, or our non-invasive surrogate of RV afterload may not be sensitive enough to detect elevations during exercise.

Downregulation and/or desensitisation of the cardiac beta-adrenergic receptors secondary to prolonged catecholamine exposure has been proposed as a mechanism for exercise-induced cardiac fatigue in the right and left ventricle. In support of this, human studies have consistently demonstrated that the cardiovascular response to a beta-adrenergic agonist (dobutamine) is reduced following a period of endurance exercise [2, 10, 12, 37], even under parasympathetic blockade [12]. As such, exercise-induced reductions in beta-adrenergic receptor activity may explain the decline in RV function following endurance activities. However, it has also been consistently demonstrated that endurance-exercise induced fatigue affects the right ventricle more (or sooner) than the left ventricle. In studies assessing the endurance-exercise response of both ventricles, left ventricular function remains normal whilst right ventricular function has declined [5, 15, 30]. It is unclear why beta-adrenergic desensitisation/downregulation would affect the RV more than the LV, unless receptor expression or density differs between ventricles. Human right and left ventricles (albeit donated by patients receiving a transplant following severe cardiomyopathy) were shown to contain a similar number of beta-adrenergic receptors and similar distribution of beta-1 and beta-2 subtypes [3]. As such, a beta-adrenergic mechanism may not underlie RV dysfunction following endurance exercise.

Alternatively, an overload-induced inflammatory response has also been suggested as a potential mechanism for RV dysfunction. The increase in certain inflammatory markers (TNF-alpha, IL-12, IL-1 beta) after endurance exercise was correlated with indices of cardiac damage (troponin, B-type natriuretic peptide) and was greater in participants showing RV dysfunction versus those that did not [18]. However, these data do not demonstrate causation, and further work is required to establish that endurance exercise does not simply drive both the inflammatory response and RV dysfunction via separate mechanisms.

### RV function during exercise

The lack of observable change in RV function during exercise in the current study is intriguing, given that we found reduced RV function in the post-exercise data and that others report altered RV function following exercise durations much shorter than 6-h [28, 29, 31]. Cycling was chosen as the exercise mode for the current study given its practical advantages for in-exercise echocardiography, although it has significantly lower energy demands than running, rowing and triathlon [27] and this may explain the maintenance of RV structure and function during exercise. However, in-exercise indices of RV work (TAPSE, S’, A’) increased as expected and in line with previous reports in athletic populations [14, 23, 34]. Thus, the current cohort did not show a lower-than-expected response to endurance exercise. Pre-event training is another factor that could explain the lack of in-exercise alteration to RV function. The magnitude of RV dysfunction after endurance exercise is inversely related to the amount of pre-endurance-event training, such that RV dysfunction was greater in those completing less training [28]. As such, a high level of pre-study training relative to the study exercise stimulus could have prevented observable changes to RV function in the current cohort. However, the participants were completing a comparable level of training as in other athletic cohorts and none had competed in an endurance event for 2 weeks prior to the study. Alternatively, participant position during echocardiographic assessment completed whilst cycling may impact measurement of RV function. Few studies have assessed RV structure, function, or PAP during exercise in an upright position and the effect of gravity on these parameters remains to be determined. Indeed, the previous studies estimating PAP during exercise have done so with participants in a supine or semi-supine position, and the effect of modifying body position when obtaining the tricuspid regurgitant signal during exercise is unknown. Assessment of PASP is more accurate using agitated saline and further work using this technique is required to establish the response of the pulmonary circulation across a range of exercise modes, intensities, and durations to determine the relative afterload placed on the RV during exercise and the consequent acute structural and functional adaptations. Furthermore, studies using echocardiography to investigate RV exercise-induced cardiac fatigue have assessed participants prior to and on completion of exercise in a supine position and not considered the effect of body position on cardiac structure and function. Most exercise is undertaken in an upright position and to truly understand the effects of exercise on cardiac structure and function, it is pertinent for assessments to be undertaken in the exercising body position to ensure that loading conditions are relevant for in-exercise assessments.

### Limitations

The small sample sized included in the current study likely influences statistical power and may, in part, explain the lack of statistical change evident in the in-exercise data. The use of a semi-supine cycle ergometer may improve image quality and afford the use of speckle tracking to assess athletes during exercise and allow the assessment of RV and LV function using a superior technique and in multiple planes. Repeated assessments of PAP during exercise would confirm whether PAP is elevated during exercise when assessed using non-invasive TR velocity-based measures. The use of 3D echo would improve structural assessment; however the frame rates are not sufficient to allow valid assessment of function especially during exercise at higher heart rates. Finally, the study did not assess LV function during or after exercise, limiting the interpretation of LV-RV dynamics.

### Conclusion

A 6-h cycling exercise bout at 75% maximum heart rate results in RV dilation and reduced RV function, however this was only evident post-exercise, with normal RV structure and function maintained during exercise. There was no evidence of increased PAP during exercise, suggesting that the RV is not placed under a higher afterload during prolonged cycling. As such, endurance exercise-induced RV dysfunction and dilation does not appear to be driven by an elevated RV afterload. Further work is needed to determine the mechanisms underlying RV dysfunction after endurance cycling.

## Data Availability

Datasets generated in the study are not publicly available to protect participant privacy but are available from the corresponding author upon reasonable request.

## Abbreviations

HR: Heart rate
PAP: Pulmonary artery pressure
PASP: Pulmonary artery systolic pressure
PLAX: Parasternal long axis view
RV: Right ventricle
RV A’: Right ventricular late diastolic myocardial tissue velocity
RV E’: Right ventricular early diastolic myocardial tissue velocity
RV S’: Right ventricular systolic myocardial tissue velocity
RVAd: Right ventricular diastolic area
RVAs: Right ventricular systolic area
RVD: Right ventricular dimension
RVFAC: Right ventricular fractional area change
RVOT: Right ventricular outflow tract
RVSp: Right ventricular systolic pressure
TAPSE: Tricuspid annular plane systolic excursion
TDI: Tissue Doppler imaging
VO_2_ max: Maximal oxygen consumption rate
